# The incidence of malaria in travellers to South-East Asia: is local malaria transmission a useful risk indicator?

**DOI:** 10.1186/1475-2875-9-266

**Published:** 2010-10-04

**Authors:** Ron H Behrens, Bernadette Carroll, Urban Hellgren, Leo G Visser, Heli Siikamäki, Lasse S Vestergaard, Guido Calleri, Thomas Jänisch, Bjørn Myrvang, Joaquim Gascon, Christoph Hatz

**Affiliations:** 1 Travel Clinic, Hospital for Tropical Diseases, Mortimer Market, London, WC1E 6JB, UK; 2 London School of Hygiene & Tropical Medicine, Clinical Research Unit, London; 3 Karolinska Institute, Karolinska University Hospital Huddinge, Department of Infectious Diseases, Stockholm, Sweden; 4 Leiden University Medical Centre, Department of Infectious Disease, Section Travel Medicine, Leiden, Netherlands; 5 Helsinki University Central Hospital, Department of Medicine, Division of Infectious Diseases, Helsinki, Finland; 6 National Malaria Reference Laboratory, Statens Serum Institut, Copenhagen, Denmark; 7 Travel Medicine Unit, Division of Infectious Diseases, "Amedeo di Savoia" Hospital, Torino, Italy; 8 Section Clinical Tropical Medicine, Department of Infectious Diseases, University Hospital, Heidelberg, Germany; 9 Oslo University Hospital, Ullevål, Department of Infectious Diseases, Oslo, Norway; 10 Dept of Travel Medicine, Division of Infectious, Barcelona Centre for International Health Research (CRESIB), Hospital Clinic, Barcelona, Spain; 11 Medical Department, Swiss Tropical and Public Health Institute, Basel, and Division of Communicable Diseases/Institute for Social and Preventive Medicine, University of Zurich, Zurich, Switzerland

## Abstract

**Background:**

The presence of ongoing local malaria transmission, identified though local surveillance and reported to regional WHO offices, by S-E Asian countries, forms the basis of national and international chemoprophylaxis recommendations in western countries. The study was designed to examine whether the strategy of using malaria transmission in a local population was an accurate estimate of the malaria threat faced by travellers and a correlate of malaria in returning travellers.

**Methods:**

Malaria endemicity was described from distribution and intensity in the local populations of ten S-E Asian destination countries over the period 2003-2008 from regionally reported cases to WHO offices. Travel acquired malaria was collated from malaria surveillance reports from the USA and 12 European countries over the same period. The numbers of travellers visiting the destination countries was based on immigration and tourism statistics collected on entry of tourists to the destination countries.

**Results:**

In the destination countries, mean malaria rates in endemic countries ranged between 0.01 in Korea to 4:1000 population per year in Lao PDR, with higher regional rates in a number of countries. Malaria cases imported into the 13 countries declined by 47% from 140 cases in 2003 to 66 in 2008. A total of 608 cases (27.3% *Plasmodium falciparum (Pf)*) were reported over the six years, the largest number acquired in Indonesia, Thailand and Korea. Four countries had an incidence > 1 case per 100,000 traveller visits; Burma (Myanmar), Indonesia, Cambodia and Laos (range 1 to 11.8-case per 100,000 visits). The remaining six countries rates were < 1 case per 100,000 visits. The number of visitors arriving from source countries increased by 60% from 8.5 Million to 13.6 million over the 6 years.

**Conclusion:**

The intensity of malaria transmission particularly sub-national activity did not correlate with the risk of travellers acquiring malaria in the large numbers of arriving visitors. It is proposed to use a threshold incidence of > 1 case per 100,000 visits to consider targeted malaria prophylaxis recommendations to minimize use of chemoprophylaxis for low risk exposure during visits to S-E Asia. Policy needs to be adjusted regularly to reflect the changing risk.

## Background

There is some evidence of declining malaria imported from Central and South America, the Indian sub continent and from West Africa [1-3]. Policy recommendations for the use of prophylaxis need to be adjusted to reflect the changing risk. Prophylaxis recommendations for travellers are published by a number of organisations and national bodies. The Swiss/German/Austrian policy group, recognized the change in travel associated risk in 2007 [4,5], and adjusted their chemoprophylaxis recommendations for travellers to the S-E Asian region but advise travellers to carry standby treatment during their journey. Other national bodies, including CDC[6], ACMP [7] Simet [8] THL, Finland[9], CMVI France [10] and WHO[11] have maintained their recommendations for the use of chemoprophylaxis to most countries where specific regional differences in incidence of malaria are reported.

Malaria prevention policy makers use a mixture of sources when deciding recommendations. Many rely on the presence or absence of malaria by species, in the country or region. Others use a combination of local malaria transmission, expert opinion and burden of disease in returned travellers to inform policy. What is not described in most policies are the methods or sources of malaria distribution, burden in returned travellers, and the expert opinion selection and qualifications to provide a transparent policy process. This means the balance between malaria threat and adverse events arising from chemoprophylaxis is unclear. The recommendations include advice on undertaking a risk assessment when selecting a prophylaxis strategy but most do not provide the epidemiological information necessary for their decision-making. This study is aimed at providing policy makers' evidence of the malaria risk faced by travellers to popular countries and regions of S-E Asia based on existing data and examine whether the current practice of deciding chemoprophylaxis policy, based on the geographical distribution of parasites, reflects the risk of travel associated malaria.

## Methods

Malaria acquired during travel was collated through case reports to national malaria surveillance bodies of 12 European countries and the USA. These countries providing data have been designated in the manuscript as source countries. The case reports do not contain details of the regions visited within countries or details of multiple countries visited during a journey. Malaria cases from France are provided through a reporting network of 120 selected hospital laboratories covering approximately half of the annual estimates of malaria cases to the Malaria National Reference Centre (CNRPalu).

Popular SE Asia countries, visited by travellers have been designated as destination countries shown in Table 1.

**Table 1:** Total numbers of visitors from source countries arriving in S-E Asia Countries

*Destination*	2003	2004	2005	2006	2007	2008
Burma (Myanmar)	60,762	54,374	53,922	71,496	35,668	26,481

Cambodia	NA	483,995	361,191	409,700	492,832	539,527

China	3,100,420	2,740,800	3,386,500	3,835,054	4,274,453	3,990,818

Indonesia	689,768	814,515	906,182	771,572	815,166	947,611

Korea	650,531	770,423	812,636	853,831	912,625	969,378

Laos	117,386	139,773	169,030	174,750	179,272	190,028

Malaysia	433,750	558,170	638,039	712,820	840,570	1,064,725

Philippines	555,879	679,011	754,805	806,865	852,828	871,581

Thailand	2,525,486	2,826,173	3,023,280	3,539,994	3,738,843	3,965,194

Vietnam	368,900	504,075	605,300	829,738	983,002	1,010,738

Source countries millions	8.5 M	9.6 M	10.7 M	12.0 M	13.1 M	13.6 M

All Foreign Arrivals millions	46.9 M	62.6 M	67.5 M	73.8 M	84.1 M	83.1 M

Data from [23,24,26-28,32,36,37]

M = millions of visits

The geographical distribution of malaria in the destination countries and the local population's malaria incidence, are based on WHO country reports from the most current published year. WHO regional offices in the Western Pacific and South East Asian Regions publish annual data provided by their member countries [12-21]. The incidence is expressed as cases (some laboratory confirmed) per 1,000 population per annum (PA). Literature reports describing the geographical distribution of malaria were used to supplement the WHO assessment of endemic transmission.

The denominator used in the study is the numbers of visitors from source countries arriving in the destination countries collated from embarkation/disembarkation cards completed for immigration and tourism purposes on arrival. Hotel registration information collated by national tourism offices provided regional visits for China, Indonesia and Malaysia. These are used to define source visitors to regions where there is a malaria risk.

## Results

A summary of the most recent malaria epidemiology assessment in the destination countries from information to regional WHO offices is examined and covers the most recent data provided by the country. The national annual incidence rates are expressed as annual parasite index (API) per 1,000 population per year. The regions and/or provinces with ongoing transmission are identified with their maximum and minimum range of API per 1,000 population per annum (PA) (Table 2)

**Table 2:** S-E Asian countries with incidence, regional transmission and total visits from source countries.

	National API Mean (range) *	**Endemic**^**+ **^**Provinces**	Source Visits	Imported cases	Mean incidence cases:100,000 visits	95% CI	Avg. duration of visit (days)
Burma (Myanmar)	2.8 (3.5-48)	10/17	302,703	31	11.80	7.69	NA

Cambodia	3.0 (3.9 -44.5)	7/24	2,287,245	45	1.16	0.76	6.6

China	< 0.01 (0.01-1.0)	3/21	21,328,045	25	0.13	0.14	17.0

Indonesia	3.8 (1-876)	17/30	4,944,814	283	3.69	2.81	13.10

Korea	0.01 (0.01-0.2)	2	4,969,424	46	0.25	0.04	NA

Laos	4 (0.78-40.0)	15/17	970,239	9	1.04	0.94	NA

Malaysia	0.2 (0.01-0.8)	2/15	4,248,074	14	0.37	0.26	NA

Philippines	0.4 (1.6-13)	25/65	4,520,969	22	0.51	0.31	23.0

Thailand	0.55 (0.4-14.5)	11/24	19,618,970	118	0.60	0.13	15.5

Vietnam	0.2 (0.6-2.7)	8/63	4,301,753	15	0.47	0.43	NA

* API of highest and lowest malaria endemic province

+ Provinces with malaria transmission and all provinces

**S-E Asian countries with incidence regional transmission and total visits from source countries**. The mean and SD is calculated from all cases and visits to the country by source visitors over the 6 period. The average duration of visit applies to the all visitors to the country based on 2008 data. NA = not available

### Cambodia

Of the 12 million population, around half a million live in forested areas with high malaria transmission. Since 2003 the incidence of malaria declined from 7:1,000 to 3:1,000 population [19]. The main provinces with malaria transmission are Battambang, Kampong Speu, Pursat, Preah Vihear, Mondukiri, Rattanakiri, Palin. (3.9-44.5:1,000 PA)[22]. Siem Reab has a population of 908,090 with a reported incidence of 3.7 cases of *Pf *per 1,000 PA. Phnom Penh receives the majority of the remaining tourists and has around 40 cases per annum occurring in its 1.4 million residents. The province of Siem Reab which includes the popular tourist complex of Angkor Wat, attracted half of the 2.3 million tourists to Cambodia in 2008 [23]. The source countries made up around 540,000 of this total (Table 1)

### Vietnam

Of the 63 provinces of Vietnam only 8 (Quang Nam, Gia Lai, Khanh Hoa, Quang Tri, Binh Phuoc, Ninh Thuan, Dal Lak, Kon Tum) are reported to have malaria with an incidence greater than >1:1,000 population per year (1.2-2.7). The nationwide incidence of malaria morbidity fell from 0.45:1,000 (2003) to 0.2:1000 PA in 2007 [17]. Of the 2.6 million tourists arriving in Vietnam in 2008, approximately 1 million were from the source countries [24].

### China

Of China's 21 provinces, the southern border provinces of Hainan, Anhui and Yunnan have a malaria transmission incidence of ~0.2:1,000 (0.13-1.0:1,000 population PA[20]. The majority of cases in Yunnan are concentrated around the international borders. Three hundred and seventy one thousand source visitors spent an average of 17 (range 11-25) nights in these three provinces during 2007[25]. China as a whole received 4 million visits in 2008 from source countries who spent an average of 17 nights during their visit [25,26].

### Thailand

The incidence of malaria has fallen from 0.86 (2003) to 0.55:1,000 populations in 2008[13]. In an exposed population of 47.4 million, around 26,000 laboratory-confirmed cases are reported of which 47% are *Plasmodium falciparum*. Eleven (Tak, Yala, Mae Hong son, Narathiwat, Songkla, Ranong, Chanthaburi, Chumporn, Kanchanabur, Petchaburi, Prachua Khiri Khan) of the 24 provinces of have an incidence > 1:1,000 PA (1.5-12.2) [22]. The Thai-Cambodia and Thai-Myanmar borders have multi drug resistant *P. falciparum *malaria. In 2007 3.7 million visitors from source countries spent an average of 14 days in Thailand, 2.5 (63%) million were independent (non-package) travellers and 40% arrived in Thailand for the first time[27].

### Malaysia

Malaria transmission is minimal on mainland Malaysia, with a country-wide incidence of 0.2:1,000 PA. The two regions with the highest incidence are Sabah and Sarawak where the 2007 rates were 0.8 and 0.5:1000 PA. Half of the cases in Sabah and 12% in Sarawak, are *P. falciparum*[18]. Malaysia received 22 million visitors [28]of which one million were from source countries 81,000 of whom visited Sarawak and 79,000 visited Sabah in 2008[29,30].

### Indonesia

Indonesia has approximately half its population living in malaria transmission areas and the highest endemicity is in the outer islands of Papua, Maluku, Nusa Tenggara, Sulawesi, Kalimantan, and Sumatra, and 17 provinces are reported to have malaria transmission. The average rate across the country is 5:1,000 PA[12]. In high endemic regions, such as Timika in Papua, the incidence has been reported to be as high as 876 per 1,000 PA[31]. Of the 5.5 million visitors to Indonesia, 947,611 originated from the source countries in 2008 remaining for an average of 13 days. Approximately half of all visitors in 2007, registered in accommodation in the endemic malaria regions for an average of 2.5 days. Of these only 67,000 were from source countries[32]

### Laos

A very recent national survey of the distribution of malaria revealed that 35% of the population lived in areas with no transmission of malaria[33]. The study was based on rapid diagnostic analysis in health centres over a three year period 2006-2009, *P. falciparum *is highly heterogeneous in the northern and central regions of the country with large areas of no transmission. In the South of the country, there is an increased risk, and there are pockets of very high and low risk and overall a lower risk of malaria for much of the population than previously described. Seven provinces, Saravane, Savannakhet, Sekong, Attapeu, Champasack, Khammouan, Phongsaly had an incidence above 1 case per 1,000 PA, with four provinces having a median of > 1 (4.7-23.5)[33]. The border regions with Vietnam had a particularly high transmission. The Lao Peoples Democratic Republic received 1.4 million visitors in 2007 of whom 180,000 were from source countries[34].

### Myanmar

More than 40 million people live in malarious areas in Myanmar, with about 4.2 million malaria cases estimated each year (about 20% of all cases in the WHO South East Asia Region), so that malaria is the most important public health problem in this country, and a priority in health planning. Most vulnerable are non-immune migrant workers, employed in rural and forest areas [14,35].

The malaria burden has steadily increased throughout the last 40 years, despite a decline between 2004 and 2005. The P. *falciparum *proportion has slowly declined, from 89% in 1988 to 75% in 2009, with *Plasmodium vivax *replacing it. There is an overall incidence of 10 cases per 1,000 population[14]. Regions with higher incidences include Magway, Mon, Sagaing, Taninthayi Division, Northern Shan State, Kachin State, Rakhine State, Chin State (6.9-48.5:1000 PA) [22] and most forested areas. Multidrug resistant *P. falciparum *is spreading, with mefloquine and quinine resistance increasing in the Thai-Myanmar border region. Of the total 100,500 tourist arrivals in Burma, 26,500 were from source countries in 2008[36].

### Philippines

Malaria in the Philippines has a countrywide incidence of 0.41:1,000 PA [15,35]. The majority of cases occur in 25 of its 65 provinces with 70% of cases reported from Palawan, Tawi-Tawi, Sulu, Apayao, Davao del Sur Cagayan (1.6-13:1000 PA) [15]. The total number of overseas visitors to the country was 2.94 million, for an average of 23 days. Visitors from the source countries made up 871,000 of this total in 2008. Over half a million of these originated from the USA [37].

### Republic of Korea

All cases are caused by *P. vivax*. The highest risk is associated with the Demilitarized Zone with most cases occurring in military personnel stationed in that area. In 2007, 2,192 cases of *Plasmodium vivax *were confirmed in the local population [16]. In 2008, 6.9 million visitors arrived in Korea of whom 969,378 were from source countries [38].

### Malaria imported from destination countries

**Figure 1:**
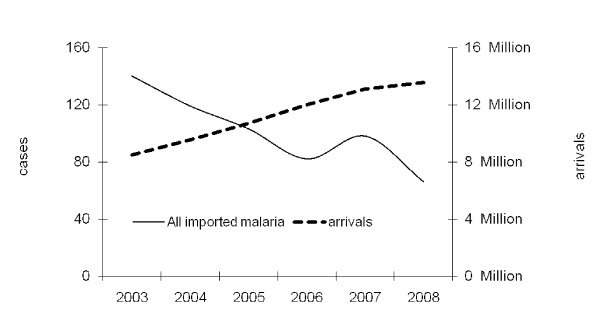
**The trend of all imported malaria from, and travel to S-E Asia between 2003 and 2008**. The trend of imported malaria and numbers of visitors to the 13 countries over 6 years. Malaria cases fell by 47% while visits to the region increased by 60%

Over the six-year period a total of 608 cases of malaria were included in the study reported by the source countries (Table 3). Table 4 details the malaria species with, *P. falciparum *(166), *P. vivax *(349), *Plasmodium ovale *(11), *Plasmodium malariae *(5) and *Plasmodium knowlesi *(3) and not-specified (65). The total number of malaria cases range from 140 cases in 2003 to 66 cases in 2008 (47% reduction) as reported by source country surveillance bodies, detailed in Table 5. *Plasmodium falciparum *constituted 30.3% of identified *Plasmodium *species during the study period. Table 6 shows all the malaria cases by county of probable acquisition by year reported. The national mean endemic rates of malaria transmission are detailed in Table 2. This also shows the range of rates from lowest to highest in endemic provinces or regions where populations are living under risk of malaria. Figure 1 shows the trend of imported malaria during the study period.

**Table 3:** All malaria cases reported by source countries and country of likely acquisition 2003-2008.

	Burma (Myanmar)	Cambodia	China	Indonesia	Korea	Laos	Malaysia	Philippines	Thailand	Vietnam	Total
Belgium	0	0	0	9	0	0	1	0	2	0	12

Denmark	0	1	1	8	0	0	1	0	4	0	15

Finland	0	1	0	0	0	0	1	0	5	0	7

France	4	12	0	34	0	2	3	4	12	2	73

Germany	7	6	0	37	2	1	2	2	20	3	80

Italy	2	0	10	21	0	0	2	0	10	1	46

Netherlands	0	2	0	61	0	0	0	3	4	0	70

Norway	1	0	1	1	0	0	0	1	3	0	7

Spain	0	1	0	4	0	0	0	2	2	0	9

Sweden	1	2	0	16	0	1	1	0	11	0	32

Switzerland	1	2	1	27	1	2	0	0	7	2	43

UK	2	6	2	16	2	1	1	2	9	0	41

USA	13	12	10	49	41	2	2	8	29	7	173

**Total**	**31**	**45**	**25**	**283**	**46**	**9**	**14**	**22**	**118**	**15**	**608**

Cases were collated through national surveillance system in the source countries.

**Table 4:** Malaria species in source countries by year of report

Species	2003	2004	2005	2006	2007	2008	Total
*P. falciparum*	47	34	24	22	22	17	166

*P. vivax*	82	63	61	41	60	42	349

*P. ovale*	2	3	3	1	1	1	11

*P. malariae*	1	1	1	1	1	0	5

*P. knowlesi*	0	0	0	0	2	1	3

unspeciated plasmodium	7	18	12	14	10	4	65

mixed cases	1	0	2	3	2	1	9

Laboratory diagnosed species as reported to respective surveillance bodies.

**Table 5:** Numbers of malaria cases reported by source countries.

	2003	2004	2005	2006	2007	2008	Total
Belgium	5	4	1	0	0	2	12

Denmark	2	6	1	2	2	2	15

Finland	0	0	1	0	4	2	7

France	17	17	7	11	10	11	73

Germany	24	16	14	11	5	10	80

Italy	14	6	12	5	4	5	46

Netherlands	19	22	16	5	5	3	70

Norway	0	4	1	2	0	0	7

Spain	1	3	1	2	0	2	9

Sweden	8	7	6	4	6	1	32

Switzerland	8	7	10	5	5	8	43

UK	13	7	4	8	5	4	41

USA	29	20	29	27	52	16	173

Total	140	119	103	82	98	66	608

**Table 6:** All travel cases by country of acquisition by year of report.

Country malaria acquired	2003	2004	2005	2006	2007	2008	Total
Burma (Myanmar)	5	5	4	4	9	4	31

Cambodia	16	6	6	9	4	4	45

China	12	4	3	1	2	3	25

Indonesia	63	63	58	38	38	23	283

Korea	10	9	4	5	16	2	46

Laos	3	2	2	0	1	1	9

Malaysia	2	2	5	1	3	1	14

Philippines	5	4	2	6	4	1	22

Thailand	20	20	16	17	18	27	118

Vietnam	4	4	3	1	3	0	15

Total	140	119	103	82	98	66	608

### Malaria incidence in visitors to destination countries

The malaria incidence is expressed as malaria case(s) per 100,000 visits with the mean for the six-year period. In destination countries where regional data of visits were available, the risk was assessed for the endemic region. Table 2 details the national incidence of all malaria using all species reports, and visits from source countries. The risk of malaria to endemic regions of Malaysia (Sabah & Sarawak) China (Yunnan, Hainan Anhui) and Indonesia (17 provinces) where transmission is regional was examined. Assuming that all cases of malaria reported were acquired in these endemic regions; in China during 2008, (371,000 visitors), there were three cases reported in travellers, in Sabah and Sarawak (161,000 visitors) there was a single case. Indonesia has the most cases reported, with 16 cases of *P. vivax *and two cases of *P. falciparum*, four other species and an estimated 67,000 visitors to the endemic provinces, based on hotel and guest house statistics [29,30]. The mean risk of malaria over the six-year period is shown in Table 2 as cases per 100,000 visits. Four countries, Burma and Indonesia, Cambodia, and Laos had a six-year average incidence of > 1:100,000 visits. However none of the other countries over the six-year period achieved a rate greater than one case per 100,000 visits and in both Cambodia and Laos during 2007-2008 rates had fallen below this level.

### Visits to destinations countries

The use of national immigration and tourism statistics has enabled a more precise estimate of travellers entering destination countries who may be exposed to malaria and provides a denominator for malaria acquired in these countries. The immigration data are detailed by the country of residence of the visitor, and from some countries the duration, reason for visit and province/city visited and type of accommodation is collected. Table 1 and Table 7 describes the increasing numbers of travellers by source countries arriving at their destination country who represent 7.25% of all foreign arrivals. The 13.6 million visitors in 2008, was an increase of 60% from the totals in 2003 (Table 1 and Figure 1).

**Table 7:** Arrivals by year from Source countries to S-E Asian countries as reported through their immigration, tourist collection and statistical reporting systems.

Departures from:	2003	2004	2005	2006	2007	2008	Totals
Belgium	92,451	101,423	92,646	200,495	216,401	215,089	0.9 Million

Finland	88,148	99,676	110,563	159,881	205,039	217,571	0.9 Million

Denmark	128,087	141,446	155,272	199,720	225,323	248,520	1.1 Million

Norway	113,098	123,354	135,386	220,722	237,990	264,597	1.1 Million

Spain	74,786	109,265	104,236	308,049	344,391	313,133	1.3 Million

Switzerland	212,284	256,911	266,936	319,966	335,341	341,797	1.7 Million

Italy	244,584	351,081	412,932	479,058	528,072	517,281	2.5 Million

Sweden	329,012	391,600	423,353	561,225	665,212	681,872	3.1 Million

Netherlands	370,878	434,818	504,335	607,181	664,011	729,697	3.3 Million

France	2,066,956	790,388	1,099,828	1,192,356	1,367,880	1,443,833	8.0 Million

Germany	934,869	1,246,917	1,368,706	1,491,276	1,628,987	1,678,263	8.3 Million

UK	1,357,393	1,704,175	1,999,223	2,087,394	2,226,187	2,395,476	11.8 Million

USA	2,638,927	3,497,791	4,039,401	4,365,706	4,669,978	4,665,820	23.9 Million

Data from sources [23,24,26-28,32,36,37]

## Discussion

Chemoprophylaxis recommendations are decided by a number of policy groups, both nationally and internationally. Many appear to base their recommendations on the malaria transmission and activity reported in S-E Asian countries, which are often historical from 2-5 years previous. Most policy documents do not state the methodology, data sources or risk analysis used in their decision-making. The basis of risk is, therefore, presumed to be predominantly linked to local or regional transmission based on WHO reports or undeclared source(s).

The popularity of S-E Asian destinations is reflected by the volume of travel to this region. During 2008, 13.6 million travellers from the 13 source countries entered the ten S-E Asian countries, constituting less than 8% of all foreign visitors. This denominator has for the first time, been based on data collated by local immigration and tourism infrastructure and provides the most representative denominator of arriving travellers. These data, which stem from all of the countries, are annually maintained, which makes the calculation of incidence straightforward. Currently, a number of other denominator sources are used to provide number of travellers, so a limited comparison was attempted. The United Nations World Tourism Organisation (WTO) arrivals are based on ticket sales and is one of the most widely used source of numbers of travellers to a county or region. The WTO figure for arrivals in China was 240% higher than the China National Statistics Office figure in the two years 2003 and 2004 [25,26]. Another denominator source for UK travellers is the International Passenger Survey (IPS) [39] which uses a sampling method interviewing departing passengers. A comparison of the IPS and Thailand Tourism data on numbers of UK travellers to Thailand over the study period enabled a comparison between two denominator sources. The IPS estimate was half (47%-57%) the numbers of UK arrivals reported by the Tourism Authority of Thailand. This comparison suggests there are significant differences in estimates of visitor arrivals by the different sources.

Malaria surveillance reports may be imprecise with regard to the actual country of infection as travellers often visit many countries within the region. If a clinical episode develops during travel, it will not be included in the national surveillance, further reducing the accuracy of these reports. This may not be a frequent problem, as detailed by The Hospital for Tropical Diseases in Bangkok, Thailand. Bangkok is a city which provides a popular starting point for travellers to the wider region. The Hospital for Tropical Diseases treated only 11 (5 *P. vivax*.) cases of malaria in travel acquired in S-E Asian countries (three from Thailand) between 2000-2005. During that period 20.1 million European and North Americans visited Thailand alone, for an average of 14 days[27] This represents a malaria incidence of < 0.01 case per 1,000 years exposure. A number of European countries, recognizing the low incidence of malaria, recommend the carriage of standby malaria therapy for travellers to the SE Asian region in place of chemoprophylaxis[40,4]. This policy ensures quality of drug can be assured as the prevalence of counterfeit and fake anti-malarial drugs in the region is high. Early self administration allows clearance of both *P. falciparum *and primary attacks of *P. vivax *infection which will reduce morbidity in true malaria cases and current therapeutic drugs are effective with minimal side-effects. The disadvantage is that all travellers (13.6 million) in this study need to carry treatment from which very few will benefit. Most treatments will not be for malaria, but for non-specific fever [41]. The life-saving advantage of standby therapy would be marginal as 70% of malaria acquired during 2008 from S-E Asia was with the less severe *P. vivax *for which standby treatment will not eliminate latent infection. A total of 17 cases of *P. falciparum *were reported that year acquired in Thailand (six), Burma (Myanmar)(three), Cambodia, Laos and Indonesia (two cases each).

A survey of backpackers in Bangkok who were travelling throughout this region described only 22% of interviewed subjects using chemoprophylaxis. A small proportion, 15% had visited forested regions during their journey where malaria is considered a risk. Most backpackers appreciated the risk of malaria, but were poorly compliant with personal protective measures and chemoprophylaxis [42] It is, therefore, unlikely that the low malaria incidence seen in most countries is because of the use of malaria preventative measures by travellers. Within the period of the Thai study (1998-2007), the Mekong countries (6 of 10) reported a 60% reduction in the annual number of local deaths attributed to malaria, and a 25% reduction in the number of confirmed cases, with Myanmar accounting for half of all reported cases [22]. This decline in transmission fits with the decreasing numbers of cases acquired by travellers despite a 12% increase in visits to the region.

The low numbers of malaria cases acquired in the most popular countries China, Malaysia, Thailand and Vietnam suggests that these countries, including their endemic provinces, pose a minimal threat to visitors. Current chemoprophylaxis recommendations may be accurately targeting visitors to these regions and may prevent malaria. This explanation is not supported by the evidence from Switzerland, [41] and Japan [43], countries which do not recommend chemoprophylaxis, but have similar or lower numbers of malaria cases in their travellers to these areas. An alternative explanation is that travellers do not visit the malaria endemic provinces. This does not fit the travel data presented here where half of all Cambodian arrivals are to Siem Reap International airport (designated by most current guidelines, as a risk region in Cambodia) and at least 300,000 visitors per year visit China's malaria endemic provinces.

Current travellers' chemoprophylaxis policy appears to use reported transmission in a province or country of any species as an indication for chemoprophylaxis. Most policies do not describe whether transmission intensity or species prevalence influence their recommendations.

Comparing the malaria risk to travellers visiting Burma (Myanmar) and Cambodia, which have similar mean national transmission rates, the actual numbers of imported malaria cases are similar (31 and 45 respectively) but the incidence in Burma (Myanmar) is 11.8 times higher. This highlights the importance of using incidence rather than absolute cases to define risk, and that the national average does not correlate with travellers risk. The relationship between endemic malaria transmission and travel-acquired malaria has been previously examined and found a similar poor correlation between endemic malaria transmission and malaria risk in visitors to Central and South America [1].

What should policy-makers use as a proxy of malaria risk for travellers? Most countries (less so Burma and Laos) have heterogeneous transmission sub-nationally because of varied vectors and forest transmission. The reported annual API across regions in the study countries varied from < 0.01- 876 per 1,000 population PA. Only four countries had national API's >= 1:1,000 PA (Table 2). However, many malaria endemic provinces and regions had higher attack rates, in a wider national setting of low transmission. However, despite high regional rates, very low numbers of malaria imported from most countries (except Indonesia) were seen. The two countries with highest incidence in travellers were Burma (Myanmar) and Indonesia (11.8 and 3.7 cases per 100,000 visits respectively) although Thailand and Indonesia provided two thirds of all cases. Indonesia has the highest endemic transmission in its islands, and despite the highest numbers of 283 imported cases the overall national incidence is lower than that of Burma (Myanmar). Using a denominator of 67,000 visits to malaria endemic provinces of Indonesia, based on hotel room and guest house statistics[32] and assuming all malaria cases were acquired in these endemic regions, the traveller's malaria incidence would be 19 cases per 100,000 visits per year (2008) or 10 cases per 1,000 years exposed. West African rates of falciparum malaria in UK residents ranged between 10 - 52 cases per 1,000 years exposure in 2006 [3].

The threshold for withdrawing chemoprophylaxis policy may be focussed on countries with low endemic transmission (<= 1.0 per 1,000 PA) and/or a high proportion of *P. vivax *(> 70%). In areas where vivax infection predominates, suppressive chemoprophylaxis may not be the ideal option as it suppresses the first attack at best, but does not prevent latent clinical episodes. Alternatively post-exposure prophylaxis may be considered.

Policy could be focussed on the incidence of malaria in travellers returning from destination countries. Surveillance reports of imported malaria are collected by most countries as is the probable country of infection. The denominator data are accessible for nearly all countries in this region although it may not be as detailed for some European countries.

Countries with rates <= 1 case per 100,000 visits, constitute a low risk and do not justify malaria chemoprophylaxis. Countries with rates > 1 case per 100,000 visits are a more variable risk, and targeted prophylaxis for regional travel or standby therapy should be considered. This strategy will limit widespread use of chemoprophylaxis for low risk travellers and reduce toxicity. Nationality, travel itinerary and threshold of risk avoidance may differ in different countries and influence this threshold, which can be adjusted. The rationale and thresholds decided by policymakers should be transparent.

The study identifies the value of incidence in travellers as a more sensitive predictor of malaria risk for travellers, particularly where there is sub national transmission. The more precise estimates reduce overuse of chemoprophylaxis with more focussed recommendations. This would reduce the well-established risk of side effects and not inconsiderable costs of widespread use of medication. Any policy should reflect both changing local transmission, for example where focal outbreaks are recorded, and the decline in transmission following national intervention programs or parasite drug resistance changes. National or international bodies should describe the data source used for informing policy and thresholds used when recommending chemoprophylaxis for regions with low numbers of imported cases. This could lead to a consensus in recommendation from policy groups advising travellers, which would improve travellers' confidence in their prophylaxis recommendation, particularly when they make comparisons with their fellow travellers on the variety of chemoprophylaxis regimens.

## Competing interests

The authors declare that they have no competing interests.

## Authors' contributions

RHB conceived the study, prepared the original manuscript and co-ordinated the data collection. BC analysed the data. LGV HS LSV GC TJ BJ CH JC on behalf of TropNetEurop, sourced and interpreted data. All authors contributed to the drafts, read and approved the final manuscript
